# Advances in cfDNA research for pregnancy-related diseases

**DOI:** 10.3389/fcell.2025.1600532

**Published:** 2025-08-05

**Authors:** Yimei Meng, Yu Yan, Xiaoshan Yue, Yan Li, Yanxiang Mo

**Affiliations:** ^1^ Department of Obstetrics, Obstetrics and Gynecology Center, The First Hospital of Jilin University, Changchun, China; ^2^ Department of Obstetrics and Gynecology, The Second Affiliated Hospital of Harbin Medical University, Harbin, China; ^3^ Department of Gynecological Oncology, The First Hospital of Jilin University, Changchun, China

**Keywords:** cell-free DNA (cfDNA), pregnancy-related disorders, non-invasive biomarkers, preeclampsia, gestational diabetes mellitus

## Abstract

Circulating cell-free DNA (cfDNA) comprises extracellular DNA fragments released into bodily fluids through cellular processes such as apoptosis, necrosis, and active secretion. Alterations in cfDNA concentration, fragmentation patterns, and molecular characteristics under physiological and pathological conditions, including pregnancy-related disorders, have established its value as a minimally invasive biomarker for early disease detection and clinical monitoring. Due to the availability of non- or minimally-invasive and scalable detection platforms with high sensitivity and specificity, cfDNA has emerged as a powerful tool in maternal-fetal medicine. This review provides a comprehensive overview of recent advances in cfDNA research, with an emphasis on its applications in pregnancy-related disorders. We elucidate the underlying biological mechanisms, current diagnostic and prognostic uses, analytical technologies, and the key challenges and future directions for clinical translation.

## 1 Introduction

Advances in molecular biology have not only deepened our understanding of genomic DNA, traditionally confined within the nucleus and mitochondria, but have also led to the identification of a distinct form of DNA known as cell-free DNA (cfDNA). cfDNA comprises extracellular DNA fragments that are released into circulation through various physiological and pathological processes, including apoptosis, necrosis, and active secretion. Unlike intracellular genomic DNA, cfDNA is freely detectable in body fluids such as blood, urine, and saliva (Jahr et al., 2001; Rostami et al., 2020). Since its discovery, cfDNA has emerged as a key biomarker in molecular medicine due to its ability to reflect dynamic biological states in a minimally invasive manner.

The discovery of fetal-derived cfDNA in maternal plasma in 1997 (Lo et al., 1997) marked a turning point in prenatal diagnostics. This breakthrough laid the foundation for non-invasive prenatal testing (NIPT), which analyzes maternal blood to detect fetal chromosomal abnormalities, such as trisomy 21 and 22q11.2 deletion syndrome (Dar et al., 2022; Zhang J. et al., 2024). Unlike traditional invasive procedures (e.g., amniocentesis), cfDNA-based screening significantly reduces procedural risks while enabling earlier and more accurate detection. As a result, cfDNA analysis has become an essential tool in modern prenatal care, offering a safer and more informative approach to pregnancy management.

Beyond aneuploidy screening, cfDNA analysis is now being explored for its potential in predicting and monitoring pregnancy complications such as preeclampsia, gestational diabetes mellitus, fetal growth disorders, preterm birth, and fetal overgrowth (macrosomia) (Guo et al., 2020; Guo Z. et al., 2025; De Borre et al., 2023; Wang Y. et al., 2023; Khalil et al., 2024). These advances are facilitated by cutting-edge molecular techniques, including next-generation sequencing (NGS) and digital PCR (dPCR), which enable high-resolution profiling of genetic and epigenetic features in cfDNA. Furthermore, its ability to predict placental-origin complications like preeclampsia and macrosomia early in gestation enables tailored clinical interventions, improving maternal-fetal outcomes.

With the continuous evolution of molecular techniques and growing insight into cfDNA biology, its role in managing pregnancy-related conditions is poised to expand, offering new avenues for early diagnosis, risk prediction, and individualized clinical care. This review aims to provide a comprehensive overview of current research on cfDNA in pregnancy-related diseases. Specifically, we discuss the diagnostic applications of cfDNA, the biological mechanisms underpinning its release and function, emerging technologies such as NGS and dPCR, and the clinical implications and challenges associated with its use in obstetric care.

## 2 Overview of cfDNA

Emerging research has demonstrated that cfDNA is not merely a passive byproduct of cell turnover but may also play active roles in disease progression (Ranucci, 2019; Zhang K. et al., 2024). For instance, *in vitro* and murine *in vivo* studies have indicated that cfDNA may be associated with enhanced viability, migration, and invasive behavior of tumor cells, suggesting a potential functional role in cancer progression (Filatova et al., 2024). Although these findings highlight the possibility of cfDNA contributing to disease mechanisms, further research, particularly in human models, is necessary to substantiate its causal involvement. Nonetheless, cfDNA remains a valuable biomarker for distinguishing between physiological and pathological states.

**FIGURE 1:**
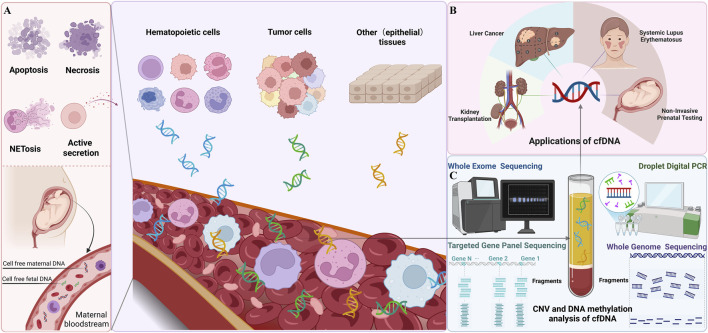
Overview of Cell-Free DNA (cfDNA) **(A)** Release Mechanisms of cfDNA The left panel illustrates the mechanisms by which cfDNA is released into the bloodstream. These include apoptosis, necrosis, NETosis, and active secretion. During pregnancy, both maternal and fetal cfDNA are present in the maternal bloodstream. **(B)** Clinical applications of cfDNA. The right panel highlights the clinical applications of cfDNA, including: Non-Invasive Prenatal Testing (NIPT): Analysis of fetal cfDNA; Liver Cancer and Kidney Transplantation: Monitoring disease and transplant status; Systemic Lupus Erythematosus (SLE): Assessing disease activity. **(C)** Detection Techniques for cfDNA (This figure was created using templates from BioRender.com).

The rapid evolution of molecular biology techniques, particularly in sequencing, amplification, and dPCR, has significantly advanced the ability to isolate and analyze cfDNA with high sensitivity and specificity. Recent advancements include the introduction of NGS technologies, which provide increased throughput and accuracy for detecting low-abundance cfDNA fragments in complex biological samples (Si et al., 2024). In addition, dPCR platforms have further enhanced the quantification of cfDNA, enabling the detection of rare genetic mutations or epigenetic modifications with unparalleled precision (Hou et al., 2023; Nazir, 2023). Importantly, cfDNA retains the genetic and epigenetic signatures of its tissue of origin, making it an ideal substrate for non-invasive diagnostics. This foundation has led to the emergence of “liquid biopsy” approaches, wherein cfDNA analysis is employed for a wide range of clinical applications. These include prenatal genetic screening, cancer detection and monitoring, assessment of therapeutic response, and prognostication (Nawroz et al., 1996; Lo et al., 1997; Zheng et al., 2012; De Vlaminck et al., 2014; Song et al., 2022). Liquid biopsy offers several advantages over traditional tissue-based methods, including reduced invasiveness, real-time disease monitoring, and the ability to capture tumor heterogeneity or fetal genomic information with minimal risk to the patient (Figure 1).

### 2.1 Release mechanisms of cfDNA

The release of cfDNA into extracellular fluids is a complex process that can occur through both passive and active mechanisms, driven primarily by cellular events such as apoptosis, necrosis, and active secretion (Mulcahy et al., 1996; Jahr et al., 2001; Stroun et al., 2001; Schwarzenbach et al., 2011). The characteristics of cfDNA, including its fragment size and molecular content, are influenced by the mechanisms through which it is released. In healthy individuals, cfDNA predominantly originates from apoptotic cells. However, minor contributions from other mechanisms, such as necrosis and active secretion, cannot be ruled out. In pathological conditions, such as cancer, cfDNA often includes contributions from both apoptotic and necrotic tumor cells, reflecting increased cell turnover and tissue damage. During apoptosis, chromatin is fragmented into nucleosomal units (∼167 base pairs), a process driven by the activation of caspase-3 and caspase-activated DNase (CAD) (Zhang and Xu, 2000; Stroun et al., 2001; Heitzer et al., 2020). The resulting nucleosomal-sized cfDNA is highly stable due to the protection conferred by the nucleosome structure, shielding it from further enzymatic degradation. In pregnancy, cfDNA is primarily derived from fetal tissues, and the placenta plays a crucial role in the release of fetal cfDNA into the maternal circulation. Syncytiotrophoblasts, which form the outer layer of the placenta, undergo a significant amount of apoptotic turnover throughout gestation, releasing fragmented DNA into the maternal bloodstream (Flori et al., 2004). This process contributes to the detectable levels of fetal cfDNA that have been widely utilized in NIPT for the detection of fetal chromosomal abnormalities. The amount of fetal cfDNA in maternal plasma increases with advancing gestational age, reflecting the increased turnover of trophoblastic cells (Ashoor et al., 2013), and is further elevated in pregnancy complications such as preeclampsia, fetal growth restriction, and placental insufficiency (Guo et al., 2020; De Borre et al., 2023; Khalil et al., 2024). In these conditions, the apoptotic release of cfDNA from the placenta is thought to be exacerbated by placental stressors, including hypoxia, inflammation, and oxidative damage.

Necrosis, another form of cell death, results in the release of larger, more irregular cfDNA fragments. This process is less regulated than apoptosis and occurs when cells undergo uncontrolled rupture, spilling their intracellular contents into the extracellular space (Jahr et al., 2001). In pregnancy-related conditions such as preeclampsia and other placental disorders, necrosis of placental tissues is common and contributes to the release of cfDNA with aberrant fragment sizes (De Borre et al., 2023). Additionally, necrotic cell death in the maternal system, such as within the endothelium or maternal tissues, may contribute to higher concentrations of cfDNA in the maternal circulation, influencing the diagnostic value of cfDNA in pregnancy complications.

NETosis, a form of programmed cell death specific to neutrophils, is also a significant contributor to cfDNA release. During NETosis, neutrophils release neutrophil extracellular traps (NETs), which consist of nuclear and mitochondrial DNA intertwined with histones and antimicrobial proteins. This process is an important component of the immune response to infection and inflammation and has been implicated in various pregnancy-related complications, such as preeclampsia, where elevated levels of cfDNA from NETs may exacerbate endothelial dysfunction and vascular damage (Margraf et al., 2008; Thierry et al., 2016; Paunel-Görgülü et al., 2017; Ronchetti et al., 2022; Cepeda et al., 2024). Inflammatory states during pregnancy, whether due to infection or autoimmune disease, may further increase cfDNA levels through the activation of NETosis in maternal neutrophils. Active secretion also contributes to cfDNA release. Extracellular vesicles (EVs), such as exosomes and apoptotic bodies, serve as vehicles for the release of DNA fragments. These vesicles, which encapsulate cfDNA, protect the DNA from enzymatic degradation and facilitate intercellular communication. EVs containing fetal cfDNA or other genetic materials have been detected in maternal blood and are thought to be involved in immune modulation These vesicles can carry not only genomic DNA but also mitochondrial DNA (mtDNA), RNA, and transposable elements, contributing to both maternal and fetal cfDNA pools (Fernando et al., 2017; Yu et al., 2021). Studies have shown that the analysis of these vesicles provides an additional layer of diagnostic information, especially in pregnancy-related diseases.

### 2.2 Biological functions of cfDNA

Traditionally viewed as a passive byproduct of cellular turnover, cfDNA is now recognized as a biologically active molecule with multifaceted roles in intercellular communication, immune surveillance, and gene regulation. Its presence in the extracellular space reflects both physiological processes and pathological perturbations, highlighting its importance in systemic regulation and host defense. One of the primary biological roles of cfDNA lies in intercellular communication. cfDNA fragments released from apoptotic, necrotic, or actively secreting cells carry both genetic and epigenetic information, including point mutations, structural variants, and DNA methylation patterns. These molecular signatures mirror the cellular source and can be internalized by recipient cells through endocytosis or receptor-mediated uptake. Once internalized, cfDNA may interact with intracellular pathways to modulate gene expression, influence cell fate decisions, or initiate stress responses. This horizontal transfer of molecular information contributes to cellular crosstalk within tissues and across organ systems, particularly under conditions of stress, injury, or disease (Dawson and Kouzarides, 2012; Bartlett et al., 2016; Shen et al., 2018; Nassiri et al., 2020). Beyond its informational content, cfDNA functions as a damage-associated molecular pattern (DAMP). When released from dying or stressed cells, cfDNA, especially that of mitochondrial or microbial origin, can activate innate immune responses. It is recognized by pattern recognition receptors (PRRs) such as Toll-like receptor 9 (TLR9) in endosomes, or cyclic GMP-AMP synthase (cGAS) in the cytosol, leading to activation of the STING pathway. These signaling cascades promote type I interferon production, pro-inflammatory cytokine release, and immune cell recruitment, implicating cfDNA in the pathogenesis of autoimmune and inflammatory conditions such as systemic lupus erythematosus (SLE) and rheumatoid arthritis (Cheng et al., 2023). cfDNA also contributes to NETs formation, a process known as NETosis. During infection or sterile inflammation, neutrophils release chromatin structures composed of DNA and histones, forming NETs that trap and neutralize pathogens. However, excessive or dysregulated NETosis can lead to tissue damage and has been associated with sepsis and thrombosis (Dawulieti et al., 2020). The cfDNA released during NETosis further amplifies inflammatory signaling and contributes to circulating DNA pools. Moreover, cfDNA plays a role in immune regulation by modulating both innate and adaptive immune responses. Its immunostimulatory properties can lead to chronic inflammation if clearance mechanisms (e.g., DNase activity and macrophage uptake) are impaired. Elevated cfDNA levels are often associated with disease activity and severity in various clinical settings. Clinically, cfDNA has emerged as a transformative biomarker. Tumor-derived cfDNA fragments reflect oncogenic alterations and are used in liquid biopsies for early cancer detection, monitoring of treatment response, and minimal residual disease assessment (McEwen et al., 2020; Zhou et al., 2021; Hallermayr et al., 2023). In maternal-fetal medicine, cfDNA has revolutionized prenatal diagnostics. Fetal-derived cfDNA circulating in maternal plasma enables NIPT for chromosomal aneuploidies and other genetic conditions, thereby reducing the need for invasive procedures and improving safety for both mother and fetus (Hoskovec and Swigert, 2018; Kwon et al., 2023). In summary, cfDNA is not merely a molecular remnant of cell death but an active participant in immune signaling, inflammation, and systemic regulation. Its diverse biological roles and clinical utility highlight its importance in both physiological and pathological processes, particularly in the field of precision medicine.

### 2.3 The role of cfDNA in early disease diagnosis

cfDNA analysis has revolutionized prenatal screening by enabling safe and accurate diagnosis of fetal and maternal conditions from a simple maternal blood sample, particularly through NIPT (Skrzypek and Hui, 2017). Fetal-derived cfDNA, originating predominantly from placental trophoblasts, becomes detectable in maternal plasma as early as 5–7 weeks of gestation and increases throughout pregnancy. NIPT enables the detection of common aneuploidies such as trisomy 21 (Down syndrome), trisomy 18, and trisomy 13 with high sensitivity and specificity. In addition to whole-chromosome aneuploidies, cfDNA can be used to screen for subchromosomal microdeletions and duplications, sex chromosome abnormalities, and in some cases, single-gene disorders through targeted sequencing approaches. Beyond fetal aneuploidy, cfDNA analysis holds promise for early detection of pregnancy-related complications, such as preeclampsia, fetal growth restriction (FGR), placental insufficiency, and preterm birth (Yu and Lo, 2024; Arbuzova and Cuckle, 2025). Studies have shown that women who go on to develop preeclampsia exhibit elevated levels of total cfDNA and altered placental-specific methylation patterns early in gestation. Similarly, aberrations in cfDNA fragment size distributions, methylation signatures, and trophoblast-specific cfDNA concentrations have been linked to FGR and spontaneous preterm labor, providing a non-invasive window into placental health and maternal-fetal interface dysfunction.

Advancements in molecular technologies have significantly enhanced the resolution and utility of cfDNA-based diagnostics. These include PCR-based approaches for precise mutation detection and NGS platforms such as targeted panels, whole-exome sequencing (WES), and whole-genome sequencing (WGS) for comprehensive genomic profiling (Chen and Zhao, 2019; Guo Y. et al., 2025). In prenatal diagnosis, methylation-sensitive assays are particularly valuable for distinguishing fetal cfDNA from maternal cfDNA, improving test accuracy for conditions with subtle genomic differences (De Borre et al., 2023). Fragmentomics, which analyzes cfDNA fragment size, end motifs, and degradation patterns, offers additional specificity by providing information on tissue of origin and disease state (Bai et al., 2024). Nucleosome positioning and chromatin accessibility profiling further enrich the biological signals derived from cfDNA, allowing inference of gene activity and cell type contributions, especially when integrated with machine learning models for predictive analytics (Ju et al., 2024).

Beyond prenatal diagnostics, cfDNA technologies are increasingly integrated into broader clinical contexts, including oncology, transplantation, and autoimmune disease management (Oellerich et al., 2021; Yang et al., 2021; Wang J. et al., 2023; Chung et al., 2024; Tong et al., 2024). However, within the scope of this section, the primary focus remains on prenatal applications, where cfDNA has revolutionized non-invasive screening for fetal aneuploidies, microdeletions, and pregnancy complications such as preeclampsia and FGR. Emerging techniques like fragmentomics and methylation profiling further enhance the specificity of prenatal cfDNA analysis, enabling early detection of placental dysfunction and maternal-fetal interface abnormalities. By aligning technological advancements with clinical needs across prenatal care and other fields, cfDNA-based approaches continue to redefine non-invasive diagnostics, offering safer and more accurate alternatives to traditional procedures.

## 3 Research on cfDNA in pregnancy-related diseases

In maternal-fetal medicine, the early identification and prediction of pregnancy complications, such as preeclampsia, fetal growth restriction, and spontaneous preterm birth, are essential for mitigating adverse maternal and neonatal outcomes. Advances in high-throughput sequencing technologies and molecular diagnostics have substantially broadened the clinical applications of cfDNA in pregnancy. Bridging the gap between technological innovation and clinical need, this section critically examines the current landscape of cfDNA research in pregnancy, with a focus on its utility for non-invasive diagnosis, risk stratification, and longitudinal monitoring.

### 3.1 Current clinical applications of cfDNA

NIPT is a revolutionary approach that utilizes high-throughput sequencing to analyze fetal cfDNA circulating in maternal plasma. This method integrates sequencing data with bioinformatic algorithms to extract fetal genetic information with high sensitivity and specificity (Lo et al., 1997; Kaiser, 2005; Lun et al., 2008). The source of fetal cfDNA is primarily apoptotic trophoblastic cells from the outer layer of the placenta. By the second trimester, fetal cfDNA constitutes approximately 10% of total cfDNA in maternal plasma (Flori et al., 2004; Ashoor et al., 2013). The biological kinetics of fetal cfDNA are distinct: it exhibits a short half-life (∼16 min) and is rapidly cleared from maternal circulation within hours postpartum, becoming undetectable after 48 h. This transient nature assures that NIPT results are not confounded by previous pregnancies or maternal genomic background (Lo et al., 1999b). The proportion of fetal cfDNA to total cfDNA, known as the fetal fraction (FF), averages between 10% and 15% during gestational weeks 10–20 (Hui and Bianchi, 2020). Fetal cfDNA fragments are characteristically shorter (∼143 base pairs) than maternal cfDNA (∼166 base pairs). Fetal-derived fragments show a sharp peak at 143 bp with periodicity of ∼10 bp, likely reflecting nucleosome phasing, whereas maternal fragments exhibit a broader distribution with less-defined periodicity (Pan et al., 2020). Traditional prenatal diagnostic techniques, such as amniocentesis and chorionic villus sampling, though accurate, are invasive and carry procedural risks including miscarriage and infection. The development of NIPT, particularly since the pivotal 2010 study by Lo et al. (2010), which used paired-end massively parallel sequencing to demonstrate that fetal cfDNA closely mirrors the fetal genome at single-nucleotide resolution, has shifted the diagnostic paradigm toward safer, non-invasive alternatives. Due to its safety, non-invasiveness, and high accuracy, NIPT is now endorsed by numerous international clinical guidelines as a first-tier screening method for fetal aneuploidies. Initially applied for detecting fetal sex, RhD status, and common trisomies (e.g., Trisomy 21, 18, and 13), NIPT has evolved with technological advancements to encompass detection of subchromosomal copy number variations (CNVs) and even pathogenic single-gene mutations (Xu et al., 2022; Zhang J. et al., 2024).

Detection of fetal cfDNA in the plasma of Rh(D)-negative mothers facilitates the determination of the fetal Rh(D) genotype. Optimal testing is typically performed after 11 weeks of gestation, when sufficient fetal cfDNA is present, significantly improving the accuracy of fetal Rh(D) testing (Chitty et al., 2014). Meta-analyses have demonstrated that this approach exhibits a sensitivity of 99.3% and a specificity of 98.4% for Rh(D) determination (Mackie et al., 2017). Several countries, including Denmark, the Netherlands, Sweden, and the United Kingdom, have integrated fetal Rh(D) testing into routine prenatal care for Rh(D)-negative pregnant women. This strategy effectively reduces the need for unnecessary administration of anti-D immunoglobulin, preventing unnecessary interventions (Wikman et al., 2012; Clausen et al., 2014). In contrast to ultrasound methods, which can typically identify fetal sex only after 12 weeks, cfDNA analysis enables the determination of fetal sex as early as the seventh week of gestation. This is accomplished by detecting Y chromosome-specific genes, such as SRY or DYS14, located on the TSPY1 region, in maternal plasma (Devaney et al., 2011). Research has shown that cfDNA-based fetal sex determination is more accurate than ultrasound. For families with a history of X-linked genetic disorders, routine NIPT for fetal sex identification can significantly reduce the rate of invasive procedures, as testing is limited to male fetuses (Hill et al., 2011; 2012; Pan et al., 2014). Additionally, non-invasive determination of fetal sex is particularly important in the management of congenital adrenal hyperplasia (CAH), a disorder primarily caused by a deficiency in fetal 21-hydroxylase, leading to masculinization of female fetuses’ external genitalia. Early identification of affected pregnancies allows for the administration of dexamethasone to suppress adrenocorticotropic hormone (ACTH) production and prevent fetal genital masculinization (Merke and Bornstein, 2005). In cases of ambiguous genitalia where ultrasound evaluation is challenging, cfDNA testing provides critical diagnostic insights. Recent studies have demonstrated that NIPT for common chromosomal abnormalities, such as trisomy 21, trisomy 18, and trisomy 13, exhibits exceptional sensitivity, with rates of 99%, 96.8%, and 92.1%, respectively (Gil et al., 2014). Although NIPT offers high sensitivity for common aneuploidies, its positive predictive value (PPV) declines for low-prevalence conditions such as rare autosomal trisomies (RATs) and subchromosomal CNVs, increasing the risk of false positives. In 2022, the American College of Medical Genetics and Genomics (ACMG) recommended the use of NIPT for the detection of common chromosomal aneuploidies, trisomy 21, trisomy 18, and trisomy 13, in both singleton and twin pregnancies, as well as for sex chromosome aneuploidies in singleton gestations. However, the ACMG currently does not support the routine use of NIPT for rare autosomal trisomies or for CNVs screening, with the exception of the 22q11.2 microdeletion syndrome (Dungan et al., 2023).

In summary, NIPT has emerged as a transformative tool in prenatal care, offering high sensitivity and specificity for common fetal aneuploidies through a non-invasive approach. Although current limitations exist for rare autosomal trisomies and subchromosomal CNVs, advances in sequencing resolution, algorithmic accuracy, and clinical validation are steadily enhancing their diagnostic scope. As these technologies mature, NIPT is expected to play an increasingly central role in comprehensive prenatal genomic screening and risk stratification.

### 3.2 Technological advancements in cfDNA analysis

Modern cfDNA testing is empowered by sophisticated molecular technologies. NGS enables the comprehensive analysis of fetal DNA fragments within maternal plasma, providing whole-genome or targeted assessments of genetic anomalies. The implementation of paired-end massively parallel sequencing, as demonstrated by Lo et al. (2010), was a pivotal step that established the fetal genome could be reconstructed from maternal blood with remarkable precision.

dPCR complements NGS by enabling highly sensitive quantification of specific cfDNA sequences, facilitating accurate detection of low-frequency variants or paternal mutations (Saito et al., 2000; Scotchman et al., 2020). These tools, when combined with bioinformatics algorithms, allow for the discrimination of fetal DNA based on size (∼143 bp vs ∼166 bp for maternal cfDNA) and methylation signatures (Hui and Bianchi, 2020; Pan et al., 2020). In cases of autosomal recessive diseases, where maternal and fetal alleles may overlap, advanced cfDNA capture platforms have been developed to resolve genotypes with higher confidence (Mu et al., 2024).

Bisulfite sequencing, which involves the conversion of unmethylated cytosine residues to uracil, is used to assess DNA methylation patterns in cfDNA, providing insights into fetal epigenetic modifications (Huang and Wang, 2019). In addition, cfDNA quantification is achieved through various methods such as dPCR, which can detect and quantify low-frequency mutations with high precision (Okada et al., 2020). Furthermore, advanced bioinformatics algorithms are used to process cfDNA sequencing data, incorporating input parameters such as fragment size, GC content, and methylation profiles, thereby enhancing the sensitivity and specificity of fetal DNA detection (Nguyen Phuong et al., 2025).

### 3.3 cfDNA and placenta-origin pregnancy complications

Emerging evidence indicates that levels of cfDNA in maternal bodily fluids, along with specific genomic features, such as distinct promoter methylation profiles, are associated with placenta-derived pregnancy complications, especially preeclampsia and macrosomia (Guo et al., 2020).

#### 3.3.1 cfDNA and preeclampsia (PE)

PE is a pregnancy-specific hypertensive disorder that affects approximately 2%–4% of pregnancies worldwide and is responsible for an estimated 46,000 maternal deaths and over 500,000 fetal and neonatal deaths annually (Magee et al., 2022). Clinically, PE is classified based on the gestational age at onset: early-onset PE occurs before 34 weeks, whereas late-onset PE occurs at or after 34 weeks of gestation. Although the precise etiology of PE remains incompletely elucidated, placental dysfunction is recognized as a key driver, particularly in early-onset forms.

Conventional diagnostic approaches, such as chorionic villus sampling, allow for direct phenotypic analysis of the placenta but are invasive and carry a non-negligible risk of miscarriage, limiting their utility in routine screening. Between gestational weeks 10 and 12, approximately 10% of cfDNA in maternal plasma originates from placental trophoblasts. This cfDNA not only reflects the fetal genome but also retains epigenetic signatures such as nucleosome positioning and DNA methylation patterns.

A seminal study by Lo et al. (1999a) reported that cfDNA concentrations in women with PE were, on average, five times higher than those in normotensive pregnant women, with elevated levels detectable before the appearance of clinical symptoms. Subsequent research by a Belgian group demonstrated that methylation patterns of cfDNA in maternal plasma at the time of delivery could differentiate PE patients from controls (De Borre et al., 2023). The same group developed a predictive model utilizing cfDNA methylation signatures measured around 12 weeks of gestation, suggesting the feasibility of using early pregnancy cfDNA profiles to stratify PE risk. Given that NIPT is now widely implemented and typically performed between 12 and 22 weeks of gestation in countries such as China, there is potential to incorporate PE risk prediction into existing cfDNA-based screening platforms. This is especially relevant for early-onset PE, where methylation alterations in placental-derived cfDNA appear to be prominent. Further supporting this approach, placental cfDNA is thought to originate, in part, from trophoblast subpopulations that express markers such as alpha-fetoprotein (AFP) and albumin (ALB), indicating a heterogeneous cellular contribution to the cfDNA pool (De Borre et al., 2023). Alpha-fetoprotein, primarily produced by the fetal liver and yolk sac, is also expressed by certain placental trophoblasts. Its concentration in maternal plasma decreases between the third and sixth months of gestation. Emerging evidence suggests that changes in AFP expression and release may influence the composition of cfDNA, and reduced AFP levels have been associated with a lower risk of preeclampsia and preterm birth. Thus, profiling AFP-related cfDNA markers in early pregnancy may offer a non-invasive means of assessing placental function and predicting adverse outcomes.

A recent study incorporated artificial intelligence to develop a predictive model for PE by integrating cfDNA metrics with clinical features collected during the first prenatal visit. Conducted as part of the SMART prospective multicenter study (n = 17,520 singleton pregnancies), the findings demonstrated an association between PE and both increased total cfDNA levels and decreased fetal fraction (Khalil et al., 2024). Moreover, a large retrospective cohort study analyzed NIPT data from pregnant women (gestational age 12–22^+6^ weeks) across four hospitals in China (2019–2021) to explore the diagnostic utility of cfDNA coverage in predicting early-versus late-onset PE (Yu et al., 2024). Gene enrichment analysis of cfDNA has identified distinct molecular signatures associated with early- and late-onset PE. Specifically, early-onset PE-related genes are enriched in Hedgehog and Hippo signaling pathways, while late-onset PE-related genes are involved in the HIF-1 and PI3K-Akt pathways. These findings suggest that cfDNA can serve as a biomarker for differentiating PE subtypes based on their underlying molecular mechanisms, highlighting its potential as a non-invasive diagnostic tool for early identification and risk stratification of PE.

#### 3.3.2 cfDNA and gestational diabetes mellitus (GDM)

GDM is characterized by glucose intolerance with onset or first recognition during pregnancy. It increases maternal risks such as preeclampsia, placental abruption, and cesarean delivery, and is associated with fetal complications including congenital anomalies, macrosomia, neonatal hypoglycemia, and respiratory distress syndrome (American Diabetes Association Professional Practice Committee, 2022). Despite advances in screening and glycemic control, the pathophysiology of GDM remains incompletely understood, limiting early predictive capabilities. Traditionally, GDM is diagnosed through second-trimester oral glucose tolerance testing (OGTT), which does not account for early molecular alterations or placental dysfunction.

Recent progress in cfDNA analysis has opened new avenues for early, non-invasive risk assessment in GDM. In addition to its diagnostic role in preeclampsia, cfDNA has shown promise in detecting GDM-related pathophysiology. GDM is associated with both pancreatic β-cell dysfunction and placental abnormalities, including increased placental size, inflammation, and impaired vascularization. These changes induce trophoblast apoptosis and necrosis, resulting in elevated levels of placenta-derived cfDNA in maternal plasma. Studies have reported significantly higher total cfDNA concentrations and fetal fraction in women with GDM, particularly in the second and third trimesters (Yuan et al., 2019; Tang et al., 2024). Hyperglycemia-induced oxidative stress and hypoxia further exacerbate cfDNA release from the placenta. Moreover, cfDNA methylation profiling has identified differential methylation in GDM pregnancies (Kenna et al., 2016). These findings suggest that cfDNA not only reflects placental dysfunction but may also serve as a molecular window into maternal-fetal metabolic health.

Complementing these mechanistic insights, a study by Wang Y. et al. (2023) employed a convolutional neural network with a non-overlapping sliding window algorithm to detect high-risk individuals as early as 12 weeks of gestation based on cfDNA signatures. Another investigation used transcription start site (TSS) profiling of 50 signature genes to differentiate GDM cases, with PRSS1, an acinar cell marker, emerging as a potential early biomarker (Tang et al., 2024). Furthermore, a neural network-based predictive model utilizing cfDNA characteristics demonstrated high predictive accuracy across validation cohorts. Altogether, these findings highlight the potential of cfDNA as a powerful, non-invasive tool for early diagnosis, mechanistic understanding, and risk stratification in GDM.

### 3.4 Clinical impact and future directions

The integration of cfDNA into prenatal care has transformed clinical practice by enabling non-invasive access to fetal genetic information. Beyond common aneuploidies, cfDNA is now used to detect pathogenic variants in single-gene disorders, especially *de novo* or paternally inherited mutations, such as those implicated in achondroplasia or thanatophoric dysplasia (Saito et al., 2000; Scotchman et al., 2020). The United Kingdom has been at the forefront of clinical adoption of NIPD for such conditions. Despite these advancements, challenges remain in detecting autosomal recessive and maternally inherited conditions, due to the confounding presence of maternal DNA. Research continues to develop more precise methods that enhance fetal genotyping accuracy, broaden mutation coverage, and reduce test turnaround time. Looking ahead, cfDNA is poised to play a greater role in early detection of a wider range of conditions, including epigenetic disorders, pregnancy complications such as preeclampsia, and even prenatal cancer detection. Emerging technologies such as long-read sequencing and machine learning-based cfDNA interpretation are expected to further refine diagnostic capabilities. When integrated with other omics data and traditional biomarkers, cfDNA analysis has the potential to revolutionize personalized prenatal care (Xu et al., 2022; Dungan et al., 2023; Mu et al., 2024).

## 4 Challenges and future prospects

Despite substantial advancements in cfDNA-based technologies, several technical and clinical limitations continue to restrict their routine application in pregnancy-related disorders. A primary challenge is the inherently low concentration of fetal cfDNA in maternal plasma, especially during early gestation, which compromises detection sensitivity and increases vulnerability to analytical noise and contamination. Compounding this issue are major technical confounders, such as the overwhelming predominance of maternal cfDNA, accounting for over 90% of total cfDNA, and hemolysis-induced release of maternal leukocyte DNA during sample collection or processing. These factors obscure fetal-derived signals and undermine assay specificity and reproducibility. Recent efforts to address these limitations include the implementation of refined pre-analytical procedures, such as the use of cfDNA-stabilizing blood collection tubes and expedited plasma isolation, aimed at minimizing maternal DNA contamination. In parallel, advanced computational strategies, encompassing fragment size profiling, methylation-based discrimination, and tissue-of-origin analysis, have been developed to enhance the resolution of fetal-specific cfDNA signatures. Nevertheless, standardization across cfDNA testing remains a critical unmet need. The lack of universally accepted protocols and clinical guidelines contributes to significant inter-laboratory variability in sample processing, data interpretation, and result reporting. This heterogeneity hampers cross-study comparability and poses a barrier to clinical integration. Therefore, the establishment of consensus standards, including quality control metrics, fetal fraction thresholds, and standardized reporting frameworks, is essential. Concurrently, efforts to define gestational age-specific reference ranges and diagnostic thresholds are gaining momentum and will be crucial for improving the clinical interpretability and utility of cfDNA-based assays. Looking ahead, integrating cfDNA analysis with emerging technologies such as machine learning and multi-omics approaches (e.g., transcriptomics, proteomics, and metabolomics) holds significant promise. These integrative frameworks could enhance predictive accuracy, enable more comprehensive biological insights, and support precision medicine applications in maternal-fetal health. Moreover, large-scale, multicenter validation studies will be vital to confirm the robustness and generalizability of these approaches for early prediction, diagnosis, and longitudinal monitoring of pregnancy complications.

## 5 Conclusion

In conclusion, cfDNA represents a transformative biomarker in the field of prenatal diagnostics, offering a non-invasive and highly informative tool for the early detection of fetal chromosomal abnormalities and maternal pregnancy-related complications. The clinical adoption of cfDNA analysis has marked a pivotal milestone in modern genomic medicine, with expanding applications in the screening, diagnosis, and monitoring of pregnancy disorders such as preeclampsia, gestational diabetes mellitus, and fetal aneuploidies. Ongoing innovations in molecular biology, sequencing technologies, and computational analysis are further enhancing the sensitivity, specificity, and clinical applicability of cfDNA assays. As these technologies mature, cfDNA testing is poised to become an integral component of precision obstetrics, supporting individualized risk assessment and early intervention strategies. Nevertheless, the realization of its full clinical potential will require continued efforts to address existing challenges, standardize methodologies, and validate findings across diverse populations and clinical settings.

## Abbreviations

cfDNA, Circulating cell-free DNA; NIPT, Non-invasive Prenatal Testing; NGS, Next-Generation Sequencing; dPCR, digital PCR; CNV, Copy Number Variation; NIPD, Noninvasive Prenatal Diagnosis; FGR, Fetal Growth Restriction; PE, Preeclampsia; GDM, Gestational Diabetes Mellitus.
